# Emerging Roles of Tubulin Isoforms and Their Post-Translational Modifications in Microtubule-Based Transport and Cellular Functions

**DOI:** 10.3390/biom16010081

**Published:** 2026-01-04

**Authors:** Aishwarya R. Nair, Nived Saroj, Ambarish Kunwar

**Affiliations:** Department of Biosciences and Bioengineering, Indian Institute of Technology Bombay, Powai, Mumbai 400076, India

**Keywords:** tubulin isotypes, tubulin isoforms, post-translational modifications, microtubule

## Abstract

Microtubules are hollow cylindrical polymers made up of tubulin. This heterodimeric protein, tubulin, exists in multiple forms: tubulin isotypes and tubulin isoforms. Distinct α- and β-tubulin genes give rise to tubulin isotypes, which differ in their amino acid sequences and cellular expression patterns. The tubulin post-translational modifications (PTMs) encode regulatory information within the microtubule lattice, modifying its biophysical characteristics and shaping interactions with motor proteins and microtubule-associated proteins. Different tubulin isotype compositions and post-translational modification patterns generate distinct tubulin isoforms. These isoforms are tissue-specific and regulate the functions of microtubules in specialized cells and cellular components such as cilia. Tubulin isoforms control cellular transport, regulate mechanosensitivity and shape the cytoskeleton, impacting the cellular functions and homeostasis. This review discusses the tubulin PTMs, including acetylation, methylation, palmitoylation, polyamination, glutamylation, glycylation, tyrosination, phosphorylation, SUMOylation, and ubiquitination, with emphasis on how isotype diversity and PTM-driven regulation together modulate microtubule behaviour, intracellular transport, and cellular functions.

## 1. Introduction

**Figure 1:**
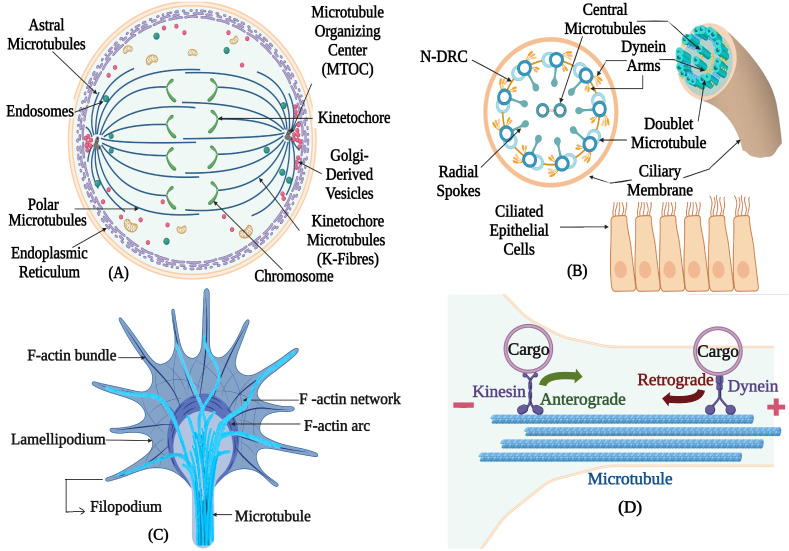
Different functions of microtubules in different essential cellular processes: (**A**) Microtubule’s role in cell division (early anaphase). (**B**) Microtubule’s structural role in cilium and ciliated epithelial cells (Scheme of a motile cilium is shown). (**C**) Organization of microtubule in the neuronal growth cone (dynamic microtubules extend from the central domain into the transition and peripheral regions to support actin–microtubule coordination and guide growth-cone steering during neurite outgrowth). (**D**) Microtubule’s role in intracellular transport along with kinesin and dynein motor proteins in axon of a nerve cell. Created in BioRender. Kunwar, A. (2025) https://BioRender.com/7z52l7p (accessed on 29 October 2025).

Microtubules are crucial elements of the cytoskeleton [1]. They perform numerous vital cellular processes (Figure 1) and functions such as intracellular trafficking, localization of cell organelles, providing structural support to the cell, regulating cell motility and cell division [2]. The microtubules are formed by highly conserved α- and β-tubulin heterodimers [3]. These α- and β-tubulin heterodimers are polymerized and arranged head-to-tail into protofilaments, forming MTs [4]. A hollow tubular structure with a diameter of 25 nm is usually formed by 13 parallel protofilaments [5]. Protofilament number varies across species; for instance, in *Caenorhabditis elegans* sensory cilia contain 15-protofilament microtubules [6]. In *Chlamydomonas reinhardtii*, ciliary axonemes exhibit the conserved eukaryotic doublet microtubule architecture, in which each outer doublet consists of a complete 13-protofilament A-tubule and an incomplete B-tubule with approximately 10 protofilaments. Although this A/B tubule asymmetry is a general feature of cilia, *Chlamydomonas* flagella display additional, doublet-specific structural specializations that generate intrinsic axonemal polarity and are linked to directional beating [7,8].

Alpha (α), beta (β), gamma (γ), delta (δ), epsilon (ϵ) and zeta (ζ) are the six tubulin family members found in eukaryotes [9]. Nevertheless, not every organism has every tubulin gene. Humans do not contain zeta tubulin. α, β and γ tubulins are abundantly found and have been identified in all eukaryotes [10]. One of the components that initiates the growth of microtubules is γ-tubulin [11]. In most cases, δ, ϵ, and ζ tubulins are specific to organisms that possess cilia, flagella, and basal bodies [12].

## 2. Tubulin Isotypes

Tubulin isotypes are a consequence of evolutionary gene duplication of ancestral α- and β-tubulin genes. The duplicated genes gradually diverged through mutations, producing isotypes that differ in their sequences and functional roles [13].

The tubulin CTT is extremely crucial as it allows the proper interaction of tubulin isotypes with a vast number of binding proteins [14]. These interactions regulate the stability, functions, and assembly of microtubules [15]. The CTT of tubulin shows extensive isotype-specific variation, arising from differences in a flexible stretch of residues that spans 17–24 amino acids at its end [16]. For instance, βIII-tubulin, predominantly found in neurons, has a unique C-terminal sequence that confers resistance to oxidative stress and promotes dynamic microtubules [17].

The expression levels of the tubulin genes are controlled in a spatial and temporal manner [18]. The number of them varies significantly between species [19] (some examples of different species with different tubulin isotypes are listed in the Table 1).

The amino acid sequences of α- and β-tubulins were fully sequenced for the first time from the mammalian brain, displaying a similarity of approximately 41% of the amino acids in the sequence [27]. About 88–95% similarity was observed in the α- and β-tubulins across different species [28]. Eukaryotic microbes were the first model systems used to study how tubulin isotypes function [25]. Two α-tubulin isotypes and a single β-tubulin isotype are found in *Saccharomyces cerevisiae* [29]. The two α-tubulin isotypes (TUB1 and TUB3) contribute differently to spindle positioning and morphogenesis, with TUB1 optimizing the Dyn1-dependent mechanism and TUB3 the Kar9-dependent mechanism [30].

In *C. elegans*, nine α-tubulin isotypes, six β-tubulin isotypes and a single γ-tubulin are present. Out of all the isotypes, MEC-12 is the only α-tubulin isotype that can be acetylated. It is found in the touch receptor neurons (TRNs), as well as other mechanosensory neurons. Specific β-tubulin isotypes are also essential for neuronal microtubule organization and dynamics [31].

Multiple α- and β-tubulin genes are found in higher eukaryotes; some isotypes are expressed universally, while others are only found in particular tissues or developmental stages. There are nine α- and ten β-tubulin isotypes in humans [9]. Among these, TUBB2A and TUBB3 are crucial for brain development, and TUBB2A is highly expressed in adult neurons [32].

### Mutations of the Tubulin Isotypes

Deletion of β-tubulin genes TUBB2A and TUBB2B, in the rodents gives rise to malformations of cortical development (MCD), which include cortical dysplasia, neuronal migration abnormalities, cerebral dysplasia, and microdysgenesis [33]. Deletion of the α-tubulin gene TUB1A in mice results in perinatal mortality with extremely dysmorphic forebrain [34].

Dominant genetic male sterility (DGMS) occurs when the widely expressed α-tubulin (αTUB84B) in *Drosophila* male germ cells is substituted with a less commonly expressed α-tubulin (αTUB85E) [22].

Disorders known as tubulinopathies are brought on by mutations in human isotypes [35]. The fundamental clinical manifestations of tubulin gene mutations include motor dysfunction, cognitive impairment, and epileptic seizures [36]. The tubulin isotypes, TUBB2A [37], TUBB2B [38], and TUBB5 mutations [39] are important and are involved in cortical development defects, whereas the disorders of axon guidance are due to TUBB3 mutations [40]. Mutations of tubulin isotypes also result in the alteration of the morphology of the blood vessels, proplatelet formation, and megakaryocyte growth [41]. Certain tubulin isotypes may also have a prognostic value in the treatment of multiple sclerosis, and some of them have been linked to the onset of cancer [42]. Several tumours, including carcinoids, observed in colorectal cancer [43] and gliomas, found in gastric cancer [44], report β-III-tubulin overexpression. Microtubules are essential in the formation of the marginal band, a subcortical ring that regulates platelet function along with their number and morphology. The nonsense mutation in the 423rd amino acid position in the CTT domain of the TUBB1, where glutamine (Q) is replaced with stop codon, causes platelet depletion, known as thrombocytopenia, causing microtubules to localize abnormally in platelets [45].

## 3. Tubulin Isoforms

Cell-specific variations in tubulin/Tubulin isoforms result from different compositions of tubulin isotypes in different types of cells [46]. Hence, Tubulin isoforms provide functional specialization for various cellular structures such as neurons, cilia, and flagella, with distinct tissue expressions [18].

Tubulin isoforms are further diversified by post-translational modifications (PTMs), which have been discussed in the subsequent Section 4 [47]. The functional diversity of microtubules is regulated through the combined expression of distinct α- and β-tubulin isotypes along with the unique PTMs [48]. Further diversification of microtubule functions is achieved by binding of microtubule-associated proteins (MAPs) that recognize tubulin isotypes and their PTMs differently, thereby affecting microtubule behaviour in specific cells [15,49].

## 4. Post-Translational Modifications of Tubulin

**Figure 2:**
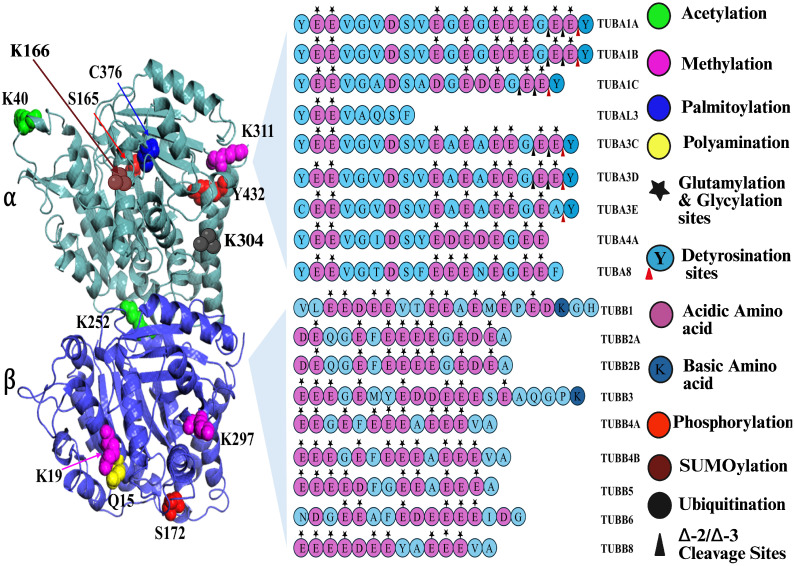
Schematic representation of all tubulin PTMs. Created in BioRender. Kunwar, A. (2025) https://BioRender.com/wc42z3w (accessed on 29 October 2025).

Tubulin protein, after its synthesis, undergoes chemical changes known as post-translational modifications. Tubulin PTMs are found within the microtubule lumen or on the MT surface [50,51]. In the C-terminal tail (CTT) of tubulin α 1A/1B, only one specific glutamic acid residue undergoes detyrosination, while the surrounding seven glutamic acid residues are potential sites for glutamylation and glycylation [52]. A schematic representation of the locations of different tubulin PTMs is shown in Figure 2.

The ‘tubulin code’ refers to the combinatorial information generated by the expression of specific α- and β-tubulin isotypes, the sequence variants (isoforms) they produce, and the PTMs added to their CTTs and internal residues [53]. A comprehensive understanding of the tubulin code can help in studying the molecular pathways and mechanisms involved in disease development.

The role of different post-translational modifications of tubulin, including acetylation, methylation, palmitoylation, polyamination, glutamylation, glycylation, tyrosination, phosphorylation, SUMOylation, and ubiquitination, in the dynamics of microtubule behaviour and cellular functions has been discussed below in detail. An overview of all tubulin PTMs with chromosome locations and function of enzymes involved is shown in Table 2.

### 4.1. PTMs of Tubulin in MT Lumen and on Surface

#### 4.1.1. Acetylation of Tubulin

**Modification sites:**

**Figure 3:**
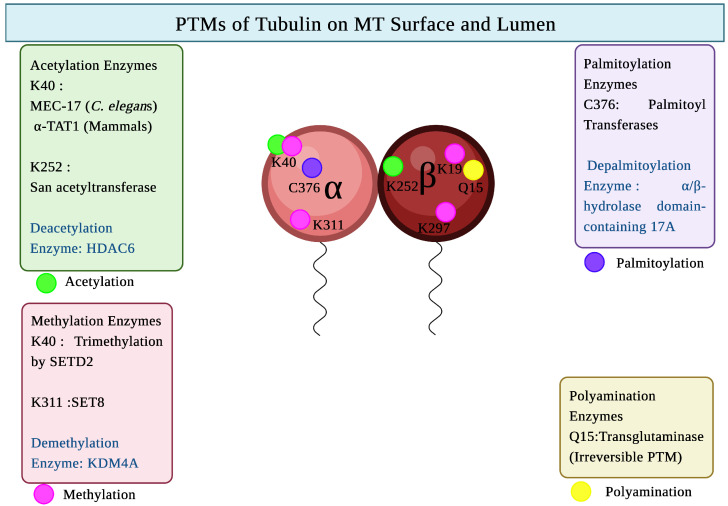
PTMs of Tubulin on MT Surface and Lumen. Created in BioRender. Kunwar, A. (2025) https://BioRender.com/qb9hbr7 (accessed on 29 October 2025).

α-tubulin lysine 40 is the site for tubulin acetylation, which is present in the lumen of the microtubule [79]. In case of β-tubulin, the site for acetylation is lysine 252 [80] (Figure 3).

**Enzymes involved:**

Alpha tubulin acetyl transferase 1 (αTAT1) is the enzyme that carries out α-tubulin lysine 40 acetylation in mammalian cells [79]. β-tubulin lysine 252 is catalyzed by San acetyltransferase.

αTAT1 is a mammalian ortholog of MEC17 found in *C. elegans* [81]. αTAT1 uses surface diffusion to scan MTs in both directions within the lumen efficiently, acetylating lysine 40 in a stochastic manner [82]. Although several enzymes, including GCN5 (General Control Non-repressible 5), ELP3 (Elongator complex protein 3), and ARD1-NAT1 (N-terminal acetylation complex), may acetylate tubulin, the significant acetylase enzyme is αTAT1/MEC17 [83]. The abolition of the action of αTAT1/MEC17 hinders MT acetylation in different models, but the αTAT1’s substrate specificity and control are yet to be explored [84].

A cytoplasmic member of the HDAC (histone deacetylase) family, HDAC6 (histone deacetylase 6), is responsible for the tubulin deacetylation. HDAC6 plays a significant role in ciliary disassembly [85]. Different protein substrates for HDAC6 have been analyzed, including α-tubulin, cortactin, Hsp90, and peroxiredoxin [86]. SIRT2 (Sirtuin 2), a class III NAD+-dependent HDAC, also functions as a tubulin deacetylase [87].

**Cellular Functions:**

This PTM remains conserved from protists to mammals and serves a wide range of regulatory purposes [6,83]. It is crucial for cell migration and structure, influencing cellular responses to stress and contributing to various physiological processes, including autophagy and mechanosensitive adhesion [88]. Tubulin acetylation helps the microtubules to repair themselves during mechanical damage, and the accumulation of acetylated residues is an indicator of the age of the microtubules [89]. This increases microtubule flexibility and stability [50]. Tubulin acetylation promotes focal adhesion dynamics, facilitating the delivery of integrins and contributing to the polarized functions necessary for effective cellular movement [90].

The mechanical behaviour of microtubules is modified by tubulin acetylation, by reducing the molecular interactions between the protofilaments of microtubules [91]. Tubulin acetylation enhances kinesin-1-mediated transport by promoting kinesin-1 processivity and load-bearing capacity during cargo transport [92]. This may influence motor protein preference, enabling specific interactions with acetylated microtubules for efficient directional cargo delivery [93]. This modification differentially impacts organelle positioning, facilitating displacement of centrosomes, mitochondria, and vimentin, while not affecting Golgi or endosomes [94]. Acetylated microtubules provide a scaffold for motor proteins, influencing transport dynamics and neuronal morphology, particularly in neurodegenerative conditions such as Parkinson’s disease, where acetylation levels are reduced [95]. Tubulin acetylation is also involved in impaired vesicle trafficking, which is observed in Huntington’s disease [96].

This PTM facilitates the transition to three-dimensional polarity in epithelial cells, supporting cargo transport towards the apical domain. During transfection, microtubule-based transport is essential for plasmid trafficking [97]. Hence, increased tubulin acetylation leads to faster plasmid nuclear localization and greater gene transfer, thereby playing a crucial role in gene therapy [98]. Defects in tubulin acetylation result in disruption of the microtubule-based transport, leading to neuronal loss as observed in Familial dysautonomia (FD), which is a neurodevelopmental disease [99].

Acetylation at the ϵ-amino group of lysine 40 of α-tubulin was initially identified in *Chlamydomonas* flagella [54]. While the majority of mammalian α–tubulin isotypes (human TUBA8 is an exception) have highly conserved lysine 40, but the α–tubulin isotypes across the species, do not exhibit the same degree of acetylation [100]. In *Tetrahymena* or *Chlamydomonas*, the lysine 40 residue of α-tubulin is not required for survival, but it seems to be important in vertebrates [101].

In the *C. elegans*, the deletion of the MEC17 (orthologue of αTAT1), damages the sensitivity to touch and disrupts the structure as well as the organization of the microtubules in the touch receptor neurons [102]. In the zebrafish, loss of the MEC17 results in developmental defects such as decreased body and head sizes, hydrocephalus and neuromuscular defects [103]. Acetylated tubulins are mostly found in neurons and cilia in zebrafish, as found in mammals. Zebrafish MEC17 mutants exhibit a nearly total loss of lysine 40 α-tubulin acetylation in neurons, but α-tubulin acetylation is retained in cilia [82,83]. In vertebrates, the role of αTAT1 still needs to be thoroughly studied [54].

HDAC6 has a critical role in diverse cellular activities, including cell signalling and cellular migration, by deacetylation of tubulin. It is involved in the mechanisms of ciliary disassembly, but the exact mechanism of cilia regulation and control is still vague [104]. The invasive dynamics of fibroblasts and carcinoma cells can be elevated by the overexpression of HDAC6, indicating that tubulin deacetylation promotes this invasive dynamics and motility [105]. Suberoylanilide hydroxamic acid (SAHA) and trichostatin A (TSA) block HDAC1 to HDAC11, all of which are NAD-independent HDACs, and inhibit invasive motility, hence these inhibitors of HDACs could be a potential anti-cancer medication [106]. HDAC6 is considered a vital therapeutic target. The selective HDAC6 inhibitor tubastatin A, for instance, has been studied to treat degenerative neurologic disorders including Parkinson’s and Alzheimer’s, cancer, and chronic obstructive pulmonary disease, without any adverse side effects [107].

#### 4.1.2. Methylation of Tubulin

**Modification sites:**

The commonly methylated residues on α-tubulin include: lysine 40 and lysine 311 [55]. Both methylation and glutamylation are observed at the α1-tubulin E434 residue (glutamic acid residue at position 434) [108] (Figure 3).

**Enzymes involved:**

The histone methyltransferase SET-domain-containing 2 (SETD2), which regulates the H3 lysine 36 trimethylation (H3K36me3) of histones, catalyzes α-tubulin methylation at lysine 40, which is the site for tubulin acetylation as well [109]. The two domains of SETD2: SET and SRI are essential for tubulin methylation [56]. Trimethylation at lysine 40 residue can be done by SETD2 [110]. In higher eukaryotes, histone and nonhistone proteins are monomethylated by the N-lysine methyltransferase SET8/PR-Set7. SET8 methylates the α-tubulin lysine 311. A transcription factor LSF was found to enhance the α-tubulin methylation catalyzed by SET8 in vitro [55]. Another protein, SMYD3, which is a member of the SET and MYND-domain (SMYD) lysine methyltransferase family methylates the lysine 311 residue of the α-tubulin. Lysine Demethylase 4A (KDM4A) demethylates α-tubulin by binding to its core catalytic domain [111].

**Cellular Functions:**

The tubulin methylation is a vital PTM regulating the properties of microtubules. This PTM is commonly found in the spindle and midbody microtubules [56]. It has been observed that methylated α-tubulin has a lesser molecular weight than that of the unmethylated tubulin [112]. Formaldehyde makes tubulin assembly incompetent by selectively methylating one or more highly reactive groups in the tubulin dimer’s α-chain [113].

The trimethylation by SETD2 at α-tubulin lysine 40 residue is necessary for the polymerization of microtubule as well as neuronal development involving neuronal polarization and neuronal migration [114]. α-tubulin lysine 40 methylation by SETD2 regulates the cell division; any disruption in this process leads to defective cytokinesis and mitosis [109]. Hence, the absence or loss of this methyltransferase SETD2 is embryonically lethal [115]. SMYD3, which is abundantly found in the centrosome, regulates cell division, plays a vital role in spindle formation, and maintains the stability of microtubules [116].

Tubulin methylation influences microtubule dynamics and properties by modifying the microtubule track, which might affect the movement of motor proteins; however, the exact effect and mechanism are yet to be explored thoroughly [109]. Mutations in SETD2 are a reason for several types of cancers, such as kidney, lung, bladder, glioma, and leukemia [117]. SETD2 mutation is very commonly observed in cases of cell renal cell carcinoma (ccRCC) [118]. The mutation at position 1625 (R1625C), which is located within the catalytic SET domain, disrupts methyltransferase activity [56]. Deletion of SETD2 results in micronuclei, polyploidy, and defects in mitotic spindle and cytokinesis [109].

During mitosis, SET8 and H4K20me are particularly elevated [55,56]. Deletion or RNAi-mediated depletion of the enzyme SET8 results in decreased chromosome compaction and impaired replication [119]. SET8, along with H4K20me1, is necessary to enter into the mitotic phase (M phase) [120]. Additionally, SET8 mediates p53 and other substrates’ monomethylation, which suppresses p53 target genes [55]. Overexpression of SMYD3 is observed in cancers, such as ovarian cancer [121] and pancreatic cancer [122]. SMYD3 is involved in the cytoskeletal reorganization in autosomal dominant polycystic kidney disease (ADPKD) [116].

#### 4.1.3. Palmitoylation of Tubulin

**Modification sites:**

Primary palmitoylation sites are cysteine residues [57]. The major site of tubulin palmitoylation is cysteine 376 found in α-tubulin [123]. Apart from cysteine 376, other less common sites for α tubulin palmitoylation are cysteine 20, cysteine 213, and cysteine 305 [123] (Figure 3).

**Enzymes involved:**

The long-chain fatty acid palmitate is covalently bonded to cysteine residues of tubulin during tubulin palmitoylation [124]. Palmitoyl-S-acyl-transferases catalyze the tubulin palmitoylation [125]. The majority of palmitoylated proteins have one to three palmitoyl residues; however, a larger number of palmitoyl residues can also be found in some cases. Tubulin palmitoylation is a reversible PTM, and the depalmitoylation of tubulin is catalyzed by α/β-hydrolase domain-containing 17A (ABHD17A) [126].

**Cellular Functions:**

Tubulin palmitoylation alters tubulin’s polymerization competence and accessibility of cysteine residues, potentially affecting motor protein interactions and microtubule stability [127]. This modification may influence transport dynamics by promoting membrane association, and might affect the motor protein interactions as well; however, further research studies are needed to understand the exact effect and mechanism [128,129]. Tubulin palmitoylation influences membrane binding and targeting, facilitating the retention of vesicles and organelles along microtubules [127]. This modification is crucial for efficient signal transduction [129,130]. Palmitoylation of α-tubulin at cysteine 377 influences astral microtubule functions during mitosis, potentially affecting protein-protein interactions with nuclear migration proteins [131]. Disruption of the enzymes involved in tubulin palmitoylation causes defects in the spindle fibres [132]. More number of palmitoylated tubulins are found in the cells during the G1, S and G2 phases of the cell cycle as compared to the M phase [132].

It has been observed that the acetylated tubulin undergoes palmitoylation, such that these palmitoylated tubulins interact with the lipid rafts of the plasma membrane, and these interactions are essential for ciliogenesis and stabilization of microtubules. Acetylated tubulin’s interaction with ceramide is observed to be enhanced by S-palmitoylation [133]. The palmitoylated α-tubulins are involved in the formation of hydrophobic interactions between the microtubules and the intracellular membranes [130]. The non-palmitoylated tubulin can be either cytosolic or membrane-associated, whereas the palmitoylated form is always membrane-associated [57]. Tubulin palmitoylation is also involved in the cell organelle spatial organization [134]. Subcellular trafficking and microtubule interactions with membranes are modulated by tubulin palmitoylation [133]. The C376S mutant α-tubulin has an impact on the positioning of the nucleus and astral MT organization in yeast [131].

Palmitoylation regulates the mechanisms of neuronal axonal transport in neurodegenerative diseases [135]. Palmitoylation/depalmitoylation controls membrane functions linked to the induction of cell signals from the cell surface, which promote cell division and trigger apoptosis [136]. Palmitoylation and depalmitoylation are important molecular processes for selective targeting of microtubule-associated protein: MAP6, to the axons, and are involved in the regulation of neuronal polarization [126]. Increased levels of tubulin palmitoylation and Ras-related protein Rab-7a are observed in the case of prostate cancer. Palmitoylated tubulins and Rab7a promote the proliferation of cells, and are essential for transporting vesicles from the plasma membrane to lysosomes that are involved in the degradation of certain receptors for signalling. Hence, palmitoylated α-tubulin can be considered as a potential therapeutic target in the case of prostate cancer [137].

Tubulin palmitoylation is inhibited by the antimitotic vinblastine, but it is still not clear how this affects vinblastine’s effect on MT polymerization [129]. In CEM leukaemic lymphocytes, treatment with a clinically relevant low dose of vinblastine results in tubulin depalmitoylation, causing microtubule depolymerization, and this is followed by apoptosis of the leukaemic cells [134].

#### 4.1.4. Polyamination of Tubulin

**Modification sites:**

Glutamine 31, glutamine 128, glutamine 133, and glutamine 285 are the sites of polyamination in α-tubulin, and glutamine 15 is the polyamination site in case of β-tubulin [47] (Figure 3).

**Enzymes involved:**

Tubulin polyamination is the covalent addition of polyamines to the glutamine residues of tubulin by transglutaminase (TG) [138]. The tubulin polyamination adds a positive charge [139]. These transglutaminases catalyze the formation of γ-glutamyl amine bonds when polyamines or monoamines are present [140].

**Cellular Functions:**

MT stability throughout neuronal development and maturation is regulated through tubulin polyamination [141]. Polyamine levels are high in the nervous system; therefore, tubulin polyamination is abundant there [142]. Tubulin polyamination has an impact on numerous facets of brain development and aging. It is correlated with cellular morphological changes, neurite formation, brain maturation, and neuronal differentiation [143]. However, the exact distribution and roles of tubulin polyamination in neurons are still not clear [59].

The genomes of humans and mice contain eight transglutaminase genes, including intracellular and secreted forms [144]. Usually secreted, TG4 and Factor XIII play a part in blood coagulation and other extracellular processes [145]. In the CNS and PNS of mammals, TG2 activity is present [146]. TG2 is involved in the tubulin polyamination and plays a crucial role in neurogenesis [147]. Apoptosis is inhibited by TG2-induced tubulin modification [148]. Tubulin polyamination is involved in controlling MT dynamics as polyaminated tubulin promotes MT nucleation and polymerization while inhibiting MT dissociation [138]. Tubulin polyamination stabilizes the microtubule during low temperature conditions and depolymerization using calcium ions or other depolymerizing drugs [139].

Transglutaminases play a role in neuronal development as well as signalling, and are associated with neurodegenerative diseases such as Alzheimer’s, Huntington’s, Parkinson’s, and amyotrophic lateral sclerosis (ALS). TG2 is found to facilitate inflammation and stabilize misfolded protein aggregates in these neurodegenerative diseases [149].

Further studies need to be conducted to understand the exact mechanism of its effect on motor proteins, cellular transport and how elevated polyglutaminase activity and polyamine levels in MTs in the brain lead to neurodegenerative disorders [58,138].

### 4.2. PTMs on the C-Terminal Tail of Tubulin

#### 4.2.1. Glutamylation of Tubulin

**Modification sites:**

**Figure 4:**
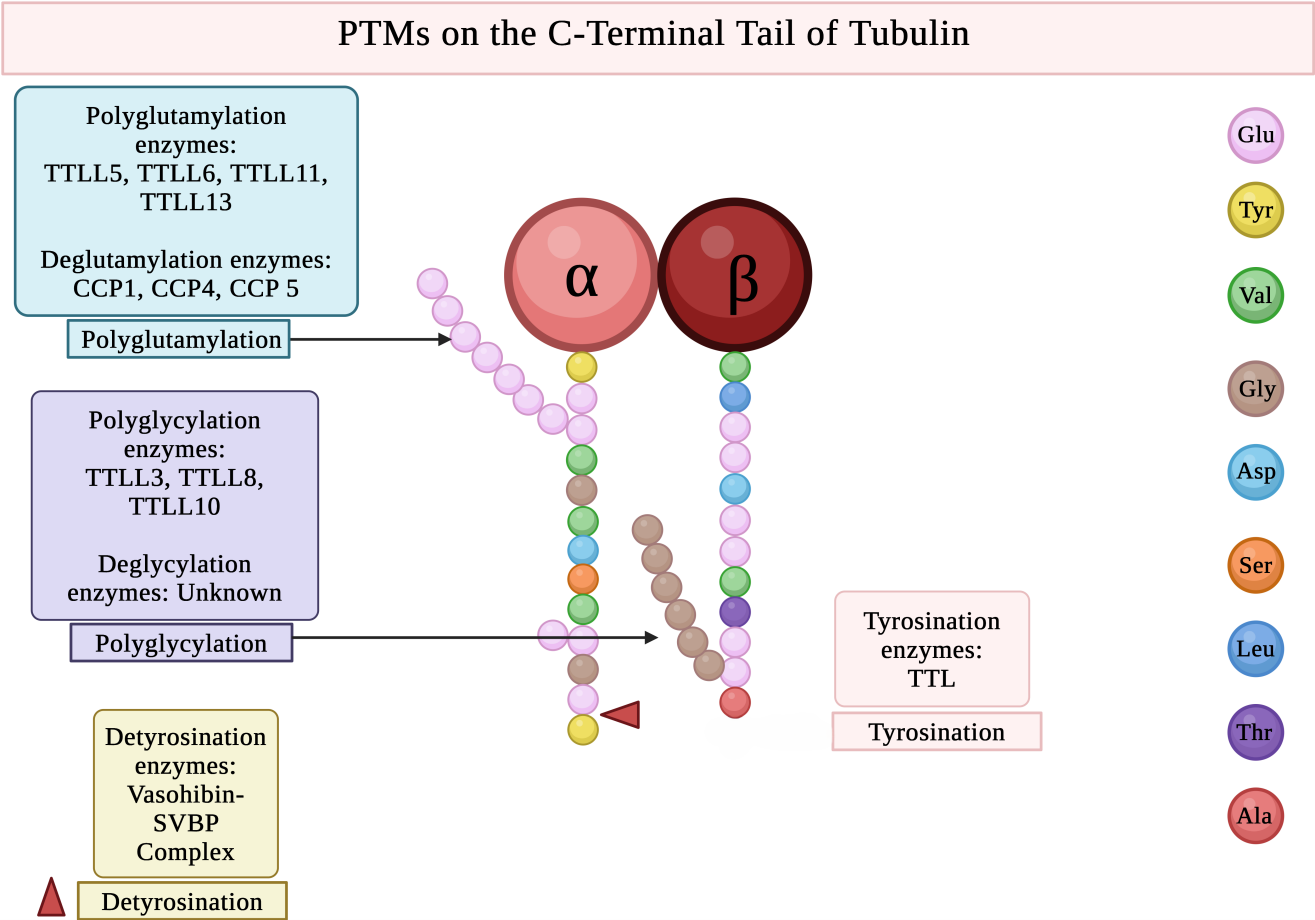
PTMs on the C-Terminal Tail of Tubulin. Created in BioRender. Kunwar, A. (2025) https://BioRender.com/5tztln0 (accessed on 29 October 2025).

This oligomeric PTM occurs when a secondary chain is added to the γ-carboxylic group of the lateral chain of a glutamate residue, at the CTT of the tubulin dimers [63]. During tubulin glutamylation, the negative charge gets added on the CTT of the α-and β-tubulins, leading to the configuration change of the microtubules at the surface [150] (Figure 4).

**Enzymes involved:**

Tubulin tyrosine ligase-like (TTLL) enzymes catalyze the PTM of glutamylation by incorporating a single glutamyl residue (monoglutamylation) or multiple glutamyl residues (polyglutamylation) to the CTT of the α- and β-tubulins [65]. The polyglutamylases: TTLL4, TTLL5, TTLL7 catalyze the initiation of the side-chain, while TTLL6, TTLL11, and TTLL13 catalyze the elongation process. TTLL5, TTLL6, TTLL11, TTLL13 are involved in the glutamylation of α-tubulin, whereas TTLL4 and TTLL7 are involved in the glutamylation of β-tubulin [63]. Tubulin glutamylases: TTLL1, TTLL4, TTLL5, TTLL6, and TTLL9 are phylogenetically conserved. *Tetrahymena* has a minimum of thirteen predicted glutamylase proteins, including all paralogous genes [151]. Cytoplasmic carboxypeptidases (CCP) are the enzymes that catalyze the tubulin deglutamylation [152].

**Cellular Functions:**

Tubulin polyglutamylation is one of the repeatedly observed PTMs in various organisms. Polyglutamylation is necessary for effective cilia motility in unicellular organisms such as *Tetrahymena* and *Chlamydomonas* [153]. The tubulin polyglutamylation pattern depends on the particular heterodimer of tubulin, the concentration of chemically modified tubulins in total, tubulin isotypes, the site of glutamylation, the size of polyglutamyl residues, and, most importantly, on the specific properties of the polyglutamylases [63,151].

Neurons are rich in polyglutamylation, with the majority of tubulin heterodimers carrying glutamate residues on the CTT with a number ranging from a single unit to six glutamate residues [52,151]. Centrioles and Axonemal MTs consist of much longer residues of glutamate [154]. During interphase, proliferating mammalian cells maintain minimal tubulin polyglutamylation; however, this modification accumulates on β-tubulin as cells progress into mitosis [155].

Tubulin glutamylation ensures proper localization and distribution of motor proteins, facilitating directional movement [156]. It is crucial for kinesin-dependent transport processes, with direct regulatory effects on kinesin-3 family motors and indirect effects on kinesin-1 activity [157,158]. Tubulin glutamylation regulates the initiation and efficiency of mitochondrial and axonal transport along microtubule tracks [159]. Increased polyglutamylation reduces overall mitochondrial motility without altering average speed or distance [160]. Axonal transport is impacted differently by glutamylation of α- and β-tubulin, indicating that glutamylation has a different effect on α- and β-tubulin [161]. Tubulin glutamylation regulates kinesin-2-mediated anterograde intraflagellar transport (IFT) by affecting signalling pathways such as Hedgehog signalling [162]. It stabilizes axonemal microtubules to support proper ciliary motility and assembly; however, excessive glutamylation can destabilize and shorten cilia, impairing intraflagellar transport efficiency [150,163].

Microtubule-severing enzymes, such as katanin and spastin, are important for the remodeling and regulation of microtubule structures [154]. Katanin preferentially localizes to glutamylated microtubules, and polyglutamylation of α-tubulin by TTLL6 enhances katanin-mediated severing [164]. The glutamylase TTLL7 further modulates katanin activity, contributing to a two-phase cleavage process [161]. Spastin activity is also regulated by tubulin polyglutamylation and exhibits a strong dependence on glutamyl side-chain length, with maximal severing occurring at intermediate chain lengths, whereas insufficient or excessive glutamylation reduces severing efficiency [61,165].

Tubulin glutamylase TTLL1 targets α-tubulin, while TTLL7 targets β-tubulin. Loss of TTLL1, but not of TTLL7, enhances mitochondrial motility in neurons; conversely, TTLL7’s activity influences β-tubulin glutamylation and modulates kinesin-based motility, without significantly affecting mitochondrial transport [166]. TTLL6-mediated polyglutamylation of axonemal tubulin is essential for normal ciliary motility, as the length of glutamate side chains specifically modulates the activity of the inner dynein arms [62]. Due to the loss of the polyglutamylase enzyme TTLL11, chromosome separation in humans gets majorly affected, and embryogenesis gets disrupted in zebrafish [167]. Depletion of this modification has been associated with neuronal homeostasis disorders and impaired ciliary motility in mice [168].

Hyperglutamylation can disrupt microtubules and lead to abnormal accumulation of MAPs [150]. Prostate cancer is associated with elevated TTLL12 expression [169]. TTLL12 has also been identified as a potential molecular marker of invasion and progression in ovarian cancer [170]. In breast cancer, TTLL4 overexpression correlates with brain metastasis and increases β-tubulin polyglutamylation, which enhances trafficking of multivesicular bodies and extracellular vesicles [171]. Elevated tubulin polyglutamylation has been associated with paclitaxel resistance in breast cancer cells [172].

In zebrafish, out of the four tubulin deglutamylases: CCP1, CCP2, CCP5, CCP6; CCP5 is the fundamental deglutamylase, involved in the cilia functioning and motility [173]. Mutations in the CCP genes can lead to neurodegenerative disorder, retinitis pigmentosa [174]. The specific mutation of the human CCP1 gene results in the infantile onset of developmental delays with prominent cerebellar atrophy [175].

#### 4.2.2. Glycylation of Tubulin

**Modification sites:**

This tubulin PTM occurs within the CTT domains and competes with tubulin glutamylation [176]. Tubulin glycylation incorporates glycine side chains on the γ-carboxyl groups of particular glutamate residues on the tubulin CTT [67] (Figure 4).

**Enzymes involved:**

A subset of tubulin tyrosine ligase-like (TTLL) enzymes, which belong to the same family as the tubulin glutamylases, catalyse tubulin glycylation. The enzymes responsible for removing this modification, however, remain unknown [153]. In mammals, TTLL3 and TTLL8 initiate monoglycylation, and the elongation step is catalyzed by TTLL10. TTLL10 gains polyglycylase activity in the presence of TTLL8 [177].

Unlike other mammals, human axonemes contain only monoglycylated tubulin due to a mutation in TTLL10 that prevents elongation, suggesting that monoglycylation may be sufficient for axonemal function [178]. TTLL3 and TTLL8 are the primary glycylases in mammalian cilia, accounting for the majority of axonemal tubulin glycylation, whereas other TTLL enzymes such as TTLL10, exhibit more limited or species-specific functions [179].

In *Drosophila*, the enzymes dmTTLL3A and dmTTLL3B function as bifunctional initiating and elongating glycylases, enabling the formation of polyglycylated tubulin [178].

**Cellular Functions:**

Tubulin glycylation is primarily associated with the microtubules of cilia and flagella [180]. It plays a central role in regulating ciliary motility and in maintaining the length and stability of primary cilia [66,181]. Across ciliated species, tubulin is mostly glutamylated, whereas glycylation is more restricted; the two modifications work together to support axoneme assembly and motility [176,181].

This modification has been examined in detail using antibodies that distinguish chain length: TAP952 detects monoglycylation, AXO49 recognises short polyglycyl chains, and PolyG identifies longer extensions [182]. An exception occurs in the Kupffer’s vesicle of zebrafish, where cilia lack detectable mono- and polyglycylation [176]. In most other ciliated tissues of zebrafish, polyglycylation is essential; its loss leads to shortened or absent motile cilia across several organs [183].

The role of tubulin glycylation is significant in the transport dynamics of axonemal dyneins and ensures proper flagellar beating [66,153]. The added glycine residues can also create a more flexible and dynamic structure on the microtubule surface, making it easier for proteins to hop between protofilaments. This results in faster diffusion across the protofilaments [184]. Tubulin glycylation modulates flagellar motility by influencing outer-arm dyneins, as it neutralizes negative charges on β-tubulin’s C-terminus.

Loss of glycylation can lead to increased acetylation, affecting microtubule properties and potentially altering transport dynamics along microtubule tracks [185]. Glycylation has been shown to increase microtubule stiffness, a change that may help axonemal microtubules withstand the mechanical loads generated during ciliary beating. This can influence how motor proteins interact with and move along the ciliary doublets [186]. Tubulin glycylation affects the efficiency and regulation of dynein-driven beating, which is important for cell movement and the generation of fluid flow [187]. Tubulin polyglycylation is also required for proper cell motility and cytokinesis [188].

Tubulin glycylation is essential for the proper function of connecting cilia in photoreceptors. In TTLL3-deficient mice, where tubulin glycylation is absent, the connecting cilium shortens, and progressive retinal degeneration occurs due to impaired transport in the cilium [176]. Male sterility linked to an axoneme assembly failure occurs in *Drosophila* when TTLL3 is depleted using RNAi [178]. Absence of TTLL3 is observed in the case of colorectal cancer [189].

In mice, tubulin glycylation is required to maintain normal ciliary structures. Loss of TTLL3 reduces the number of primary cilia in colon epithelial cells and destabilizes motile cilia in ependymal cells [65]. Because glycylation loss is accompanied by increased tubulin glutamylation, it remains important to determine whether this hyperglutamylation contributes to the ciliary defects observed in TTLL3-deficient mice [47]. In *Tetrahymena*, deletion of TTLL3 causes a mild reduction in tubulin turnover and produces slightly shorter cilia [183].

#### 4.2.3. Tyrosination of Tubulin

**Modification sites:**

The tubulin tyrosination/detyrosination is the PTM that occurs at the CTT of α-tubulin by the addition or removal of tyrosine residues, respectively [190] (Figure 4).

**Enzymes involved:**

The addition of tyrosine residues is carried out by tubulin tyrosine ligase (TTL), whereas the removal of tyrosine residues is carried out by tubulin carboxypeptidases [185]. Vasohibins (VASH1 and VASH2) are the enzymes with tubulin carboxypeptidase activity, which is required for the detyrosination of tubulin. VASH were originally identified as secretory proteins, which are induced by VEGF (vascular endothelial growth factor) during the process of angiogenesis [191].

**Cellular Functions:**

Tubulin tyrosination marks dynamic microtubules, whereas detyrosination is associated with more stable microtubule populations [192]. This cycle influences how motor proteins interact with the microtubule lattice: kinesins show higher affinity for detyrosinated microtubules [193], while cytoplasmic dynein preferentially engages with tyrosinated tracks [69]. Tyrosinated tubulin also recruits CAP-Gly (Cytoskeleton-Associated Protein-Glycine-Rich) proteins to microtubule plus ends, a process that is disrupted when tubulin is detyrosinated [194,195]. In cilia, tyrosination/detyrosination helps sort intraflagellar transport trains [196].

The balance of this modification is important for several cellular processes. Detyrosination supports kinesin-1-driven transport of APC (Adenomatous Polyposis Coli) protein to the cell cortex during cell polarization [197], enhances EB1 (End-Binding protein 1) interactions with microtubules linked to focal adhesions [198], and promotes stable kinetochore–microtubule attachments through CLASP2 (Cytoplasmic linker-associated protein 2) and NDC80 (kinetochore complex component) [70,199]. Dysregulated detyrosination delays chromosome congression by altering CENP-E (Centromere-associated protein E) movement [200]. In neurons, tyrosinated microtubules support growth cone assembly, whereas detyrosinated and acetylated microtubules stabilize neurites [68]. TTL-null neurons mislocalize CLIP170 (Cytoplasmic Linker Protein 170) and show defective neurite extension [194,201].

Species-specific differences highlight the functional diversity of this cycle: in *Leishmania*, detyrosination alters flagellar remodeling and increases kinesin-13 activity, which affects microtubule disassembly and cellular morphology [202].

Increased detyrosinated tubulin is found in several tumour types [203] and is particularly elevated in breast carcinoma [204]. Inhibition of TTL, which increases detyrosination, promotes cancer cell proliferation [205]. VASH1 and VASH2, the enzymes responsible for tubulin detyrosination, play contrasting roles in cancer progression: VASH1 inhibits tumour growth, whereas VASH2 promotes migration, metastasis, and angiogenesis [191,206]. Co-depletion of VASH1 and VASH2 reduces detyrosinated tubulin levels, highlighting their importance in tyrosination/detyrosination cycle [207]. Excessive detyrosination also contributes to microtentacle formation in breast cancer cells, facilitating metastasis [208].

Beyond cancer, excessive detyrosination contributes to cardiomyopathies such as hypertrophic cardiomyopathy, where highly detyrosinated microtubules impair cardiomyocyte contractility [209]. TTL-null mice die shortly after birth due to severe neuronal disorganization, and reduced re-tyrosination has been associated with neurodegenerative conditions, including Alzheimer’s disease [190].

### 4.3. PTMs Providing Regulatory Tags on Tubulin

#### 4.3.1. Phosphorylation of Tubulin

**Modification sites:**

**Figure 5:**
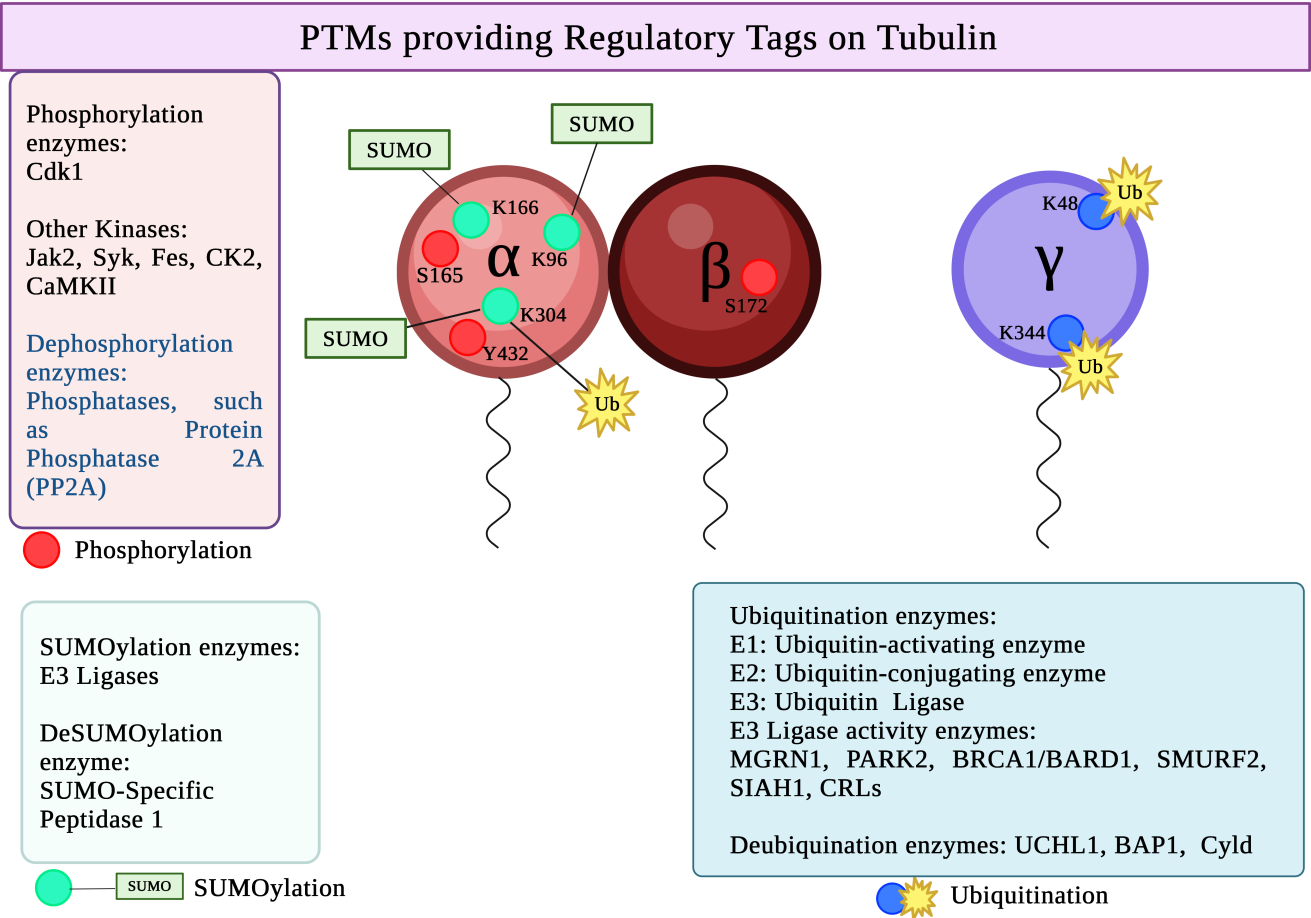
PTMs providing Regulatory Tags on Tubulin. Created in BioRender. Kunwar, A. (2025) https://BioRender.com/8pogbdn (accessed on 29 October 2025).

Serine 172 in β-tubulin is the major site of phosphorylation [71] (Figure 5). Other phosphorylation sites include serine 165, tyrosine 432 in α-tubulin, and serine 131, serine 385 in γ-tubulin.

**Enzymes involved:**

Tyrosine kinases such as Jak2 (Janus kinase 2), Syk (Spleen Tyrosine Kinase), Fes (FES proto-oncogene, tyrosine kinase), and Src (Src proto-oncogene, non-receptor tyrosine kinase), as well as serine/threonine kinases such as Cdk1 (cyclin-dependent kinase 1), CK2 (Casein kinase 2), and CaMKII (Calcium/Calmodulin-dependent protein kinase II), phosphorylate α- and β-tubulins [210]. Serine 172 of β-tubulin is phosphorylated by Cdk1 and DYRK family kinases. α-tubulin serine 165 residue gets phosphorylated by protein kinase C (PKC), and results in the elongation of microtubules and affects the motility and dynamics of microtubules [211]. Phosphorylation at the serine 131 and serine 385 of γ-tubulin is carried out by Ser/Thr kinase SadB [212].

**Cellular Functions:**

Tubulin phosphorylation was first identified on β-tubulin in microtubules of differentiated neuroblastoma cells [213].

Several kinases act directly on tubulin. Casein kinase II increases tubulin affinity for NP185 (Neuronal protein), which participates in clathrin assembly [214]. Jak2 phosphorylates tubulin on tyrosine residues [215], and pp60c-src modifies tubulin in nerve growth cones [216]. Neuronal β-III tubulin is heavily phosphorylated on serine and possibly tyrosine residues, specifically in its monoglutamylated form [217,218]. Cdk1-dependent phosphorylation at serine 172 disrupts microtubule polymerization [211], and mutations at this residue are linked to human neurological disorders [219]. Calcium–calmodulin–dependent phosphorylation modulates transport in neurons [220].

α-Tubulin is phosphorylated by insulin receptor kinase at a C-terminal tyrosine residue, and this modified α-tubulin does not incorporate into microtubules [221]. PKCα phosphorylates α6-tubulin at serine 165; the S165D phosphomimetic mutant serves as a marker for metastatic breast carcinoma [211,222].

Phosphorylation also affects ciliary proteins. DYF-5/MAK (Male Germ Cell-Associated Kinase) phosphorylates IFT-74 (Intraflagellar transport protein 74), reducing tubulin binding to the IFT74/81 complex and promoting tubulin release at the ciliary tip [72].

γ-tubulin phosphorylation contributes to centrosome and cell-cycle control. SadB modifies γ-tubulin at serine 131 to promote centrosome duplication, and phosphorylation at serine 385 regulates its localization and DNA replication fidelity [223]. γ-tubulin phosphorylation also influences mitotic progression and nuclear localization [212]. The c-Fes kinase regulates tubulin polymerization during neuronal differentiation [224].

Disease-linked phosphorylation events include DYRK1A-dependent β-tubulin phosphorylation, which affects dendritic morphology and is elevated in Down syndrome and autism [225]. Phenyl saligenin phosphate modifies phosphorylated tubulin residues in neurons and contributes to neurotoxicity [226]. Tau hyperphosphorylation disrupts microtubule interactions and is characteristic of Alzheimer’s disease [227]. Fluoxetine reduces tau phosphorylation and modifies tau–tubulin interactions in chronic stress models [228].

In immune cells, α-tubulin is tyrosine-phosphorylated in T-cells during CD3-dependent activation [229], while Syk phosphorylates tubulin dimers in B-cells, enabling interactions with MAP2 and SH2-domain proteins [230,231]. c-Abl kinase–dependent γ-tubulin phosphorylation is elevated in neurodegeneration, and its inhibition by nilotinib or bafetinib shows positive therapeutic effects in Parkinsonian models [223]. Loss of β-III tubulin phosphorylation impairs microtubule assembly [217].

#### 4.3.2. SUMOylation of Tubulin

**Modification sites:**

Lysine 96, lysine 166, and lysine 304 of soluble α-tubulin are the primary sites of SUMOylation on α-tubulin (Figure 5).

**Enzymes involved:**

Tubulin SUMOylation occurs when small ubiquitin-related modifier (SUMO) is covalently bonded to the lysine residues of the tubulin dimers. To attach SUMO molecules to target proteins, the SUMOylation process necessitates a number of coenzymes, such as ligase E3, binding enzyme E2, and activating enzyme E1 [232]. Small ubiquitin-related modifier (SUMO)-specific peptidase 1 reverses SUMOylation by removing SUMO proteins from tubulin [73].

**Cellular Functions:**

The SUMOylation of tubulin is one of the less common tubulin PTMs; this PTM closely resembles the process of ubiquitination [74]. Both SUMO and ubiquitin have a β-grasp fold in their tertiary structures, a characteristic observed in the ubiquitin protein family [233]. Despite this structural similarity, there are also some distinctions between the two molecules [234]. An additional 20-amino-acid extension at the N-terminal is present in SUMO, which makes it different from ubiquitin [235].

The size of SUMO is roughly 100 amino acids [236]. Highly conserved SUMO proteins are present in different species, ranging from yeast to mammals [237]. SUMO comes in four different isoforms in mammals: SUMO1, SUMO2, SUMO3, and SUMO4 [238]. The traditional binding of SUMO takes place at the lysine residue located within the consensus sequence (ΨKxE/D), where Ψ represents a large hydrophobic residue and x represents any amino acid [239].

SUMOylation controls the cellular localization of tubulin and is involved in several crucial cellular functions such as DNA replication, DNA repair, transcriptional control, and nuclear translocation [240]. Tubulin SUMOylation affects microtubule stability by weakening protofilament interactions; incorporation of SUMOylated α-tubulin into microtubules promotes lattice disruption and ultimately drives microtubule disassembly [73]. In addition, tubulin SUMOylation influences the organization of other cytoskeletal systems, including actin, septins, and intermediate filaments [240].

Tubulin SUMOylation may influence motor protein interactions and microtubule stability, potentially affecting transport processes. However, specific mechanisms and the extent of SUMO’s role in altering motor protein dynamics remain under investigation.

Tubulin SUMOylation is involved in neurite growth [73]. Numerous neurological conditions, such as neurodegenerative diseases, spinal cerebellar ataxia, cerebral ischemia, and epilepsy, are brought on by disruption of SUMOylation [241]. Neurotransmitter transmission and synaptic plasticity may also be impacted by tubulin SUMOylation. As a result, tubulin SUMOylation could be a potential therapeutic target for several neurological disorders [75].

#### 4.3.3. Ubiquitination of Tubulin

**Modification sites:**

The ubiquitination sites include lysine 304 in α-tubulin, lysine 48 and lysine 344 in γ-tubulin (Figure 5).

**Enzymes involved:**

Three enzymes are involved in the ubiquitination process: E1, E2, and E3, which are the ubiquitin-activating enzyme, ubiquitin-conjugating enzyme, and ubiquitin-ligase, respectively [242]. BRCA1/BARD1 form the E3 ubiquitin ligase and ubiquitinate centrosomal proteins, including the γ-tubulin [243]. Two substrates involved in the ubiquitination of the centrosome are cyclin B and Nek2, which is a cell cycle-regulated kinase [244,245].

**Cellular Functions:**

Tubulin ubiquitination plays a central role in the degradation of misfolded or defective tubulin [246]. One of the best-characterized mechanisms involves the E3 ubiquitin ligase parkin, which binds and ubiquitinates tubulin dimers.

α-tubulin ubiquitination is also required for specialized processes such as flagellar disassembly, where its disruption affects axonemal turnover [78]. Several cytosolic E3 ligases contribute to this regulation, including Mahogunin Ring Finger-1 (MGRN1) [247]. Loss of MGRN1 catalytic activity results in aberrant mitotic spindle formation through defective α-tubulin monoubiquitination, without altering β- or γ-tubulin levels or the polymerization status of microtubules [248,249]. MGRN1-dependent α-tubulin ubiquitination also maintains microtubule dynamics involved in intracellular transport; its depletion impairs mitochondrial and endosomal trafficking due to disrupted motor protein movement [249].

Several E3 ubiquitin ligases additionally regulate centrosomal protein stability to ensure proper cell proliferation and genomic integrity [250]. Dysregulated centrosome number is a hallmark of many cancers [251]. Ubiquitination of γ-tubulin is highly specific, and its perturbation disrupts spindle assembly, centrosome organization, and kinetochore attachment—processes essential for accurate chromosome segregation [252]. The BRCA1/BARD1 E3 ligase complex similarly contributes to centrosome regulation by ubiquitinating centrosomal components and restraining centrosome-driven microtubule nucleation; loss of BRCA1 function results in centrosome hypertrophy, a phenotype frequently observed in breast cancer cells [77,253].

Other E3 ligase complexes, including the Anaphase Promoting Complex (APC) and the Skp1–Cullin–F-box (SCF) complex, regulate mitosis and cell-cycle progression either directly by modulating spindle assembly checkpoints or indirectly through the turnover of microtubule-interacting proteins [254,255,256].

Parkin mutated in certain familial Parkinson’s disease cases, targets both α- and β-tubulin for degradation through predominantly hydrophobic interactions [76,257]. In models of Parkinson’s disease, protease inhibition leads to the accumulation of parkin and ubiquitinated α-tubulin in aggregated forms, indicating impaired proteostasis in dopaminergic neurons [258]. Under proteasome inhibition, parkin also localizes to microtubules and the centrosome, suggesting that MTs and MT-associated proteins may facilitate ubiquitin–proteasome activity at centrosomes [259,260].

## 5. Discussion

Understanding how tubulin post-translational modifications interact on microtubules is essential for interpreting their regulatory impact, yet current knowledge indicates that many of these relationships remain unresolved. Several modifications occur on overlapping residues or influence the accessibility of neighbouring sites, creating situations in which PTMs either compete or alter one another’s functional consequences. For example, competition between glutamylation and glycylation for the same C-terminal glutamate residues can shift the ability of motor proteins to engage the lattice, while detyrosination modifies surface recognition in a manner that affects the impact of other CTT modifications [65,164,194]. These observations suggest that PTMs interactions occur either by altering access to shared residues, changing local electrostatic properties, or modifying the conformation of regions targeted by other enzymes. What remains unresolved is how these factors collectively determine whether two modifications can coexist, whether one modification prevents another, or whether modifications occur in a defined sequence.

Although many modifying enzymes have been identified, the mechanisms underlying their spatial restriction remain unclear. It is unclear whether local factors such as small changes in lattice structure, forces generated by motor activity or cell movement, or the isotype composition, alter how modifying enzymes access and modify specific microtubule regions. These uncertainties are particularly relevant when considering how PTM patterns differ across axons, dendrites, cilia, and mitotic structures, even when the same enzymes are present [141,185,187].

Several PTMs remain insufficiently characterised and they might be significantly contributing to regulatory effects on microtubules. Polyamination, despite being one of the earliest-detected modifications, is poorly understood with respect to its dynamics and potential to influence motor progression or resistance to mechanical stress [140,142]. SUMOylation and ubiquitination of tubulin have been reported, but it is not yet known whether these modifications influence how particular tubulin molecules are removed, degraded, or replaced within microtubule networks, or how they interact with other PTMs on the same microtubule segment [240,249]. Palmitoylation is another modification whose spatial distribution and impact on membrane-proximal microtubules remain undefined [123,127]. Tubulin methylation has recognised functions in mitosis, but whether it modifies the accessibility or activity of other PTM enzymes is unknown [109,114].

Another question is whether PTMs influence microtubule behaviour mainly by changing how motors, MAPs, and severing enzymes bind to the polymer, or whether some PTMs also produce small structural alterations in the lattice itself [92,154,164]. Even minor changes in protofilament alignment or spacing could modify how motors move along the filament, how easily severing enzymes engage the lattice, or how mechanical forces are transmitted along microtubules during cell movement or cargo transport [186].

Finally, the temporal coordination of modifications remains incompletely understood. Acetylation, polyamination, glutamylation accumulate slowly on long-lived microtubules, whereas tyrosination, phosphorylation, SUMOylation, and ubiquitination respond quickly to changes in signalling or mechanical forces [138,139,192]. How these PTMs interact, particularly when competing for the same target residues, has not been established. Whether sequential modification events occur in a defined order or reflect stochastic enzyme access is a major unanswered question.

A key challenge moving forward is to determine how changes in PTM levels translate into measurable differences in microtubule behaviour. In many systems, enzymes are present throughout the cytoplasm, yet PTM patterns remain highly compartmentalised, indicating that local features of the microtubule influence which modifications accumulate and how they persist [65,185]. Understanding how patterns of isotype incorporation shape PTM dynamics will be essential for interpreting why some microtubule populations support sustained transport, whereas others remain highly dynamic. Resolving these questions will provide a better mechanistic understanding of how microtubules acquire specialised functions in different cellular regions.

## 6. Conclusions

Microtubule-based cellular functions are regulated by the combined effects of tubulin isotypes and PTMs. In-depth understanding of these regulations will help explain why changes in isotype expression or PTM patterns are linked with disorders such as neurodevelopmental disease, neurodegeneration, ciliopathies, and cancer. High-resolution structural methods, combined with live-cell measurements of modification dynamics and targeted manipulation of modifying enzymes, will be essential for determining how specific combinations of isotypes and PTMs collectively modify the microtubule properties.

## Figures and Tables

**Table 1:** Diversity in tubulin isotypes across different species.

Species	Tubulin Isotype α	Tubulin Isotype β
*Homo sapiens*	TUBA1A, TUBA1B, TUBA1C, TUBA3C, TUBA3D, TUBA3E, TUBA4A, TUBA8, TUBAL3 [20,21]	TUBB or TUBB5, TUBB2A, TUBB2B, TUBB3, TUBB4A, TUBB4B, TUBB1, TUBB6, TUBB8, TUBB8B [21]
*Mus musculus*	TUBA1A, TUBA1B, TUBA1C, TUBA4A, TUBA3A, TUBA3B, TUBA8, TUBAL3 [20]	TUBB1/ TUBB2A, TUBB2B, TUBB3, TUBB4A, TUBB4B, TUBB5, TUBB6 [20]
*Drosophila melanogaster*	TUB67C, TUB84B, TUB84D, TUB85E, TUB90E [22], refer FlyBase (https://flybase.org accessed on 29 October 2025)	TUB85D, TUB65B, TUB97EF, TUB60D, TUB56D [22], refer FlyBase (https://flybase.org accessed on 29 October 2025)
*Caenorhabditis elegans*	TBA-1, TBA-2, MEC-12, TBA-4, TBA-5, TBA-6, TBA-7, TBA-8, TBA-9 [23,24]	TBB-1, TBB-2, MEC-7, TBB-4, BEN-1, TBB-6 [23,24]
*Saccharomyces cerevisiae*	TUB1, TUB3 [25]	TUB2 [25]
*Tetrahymena thermophila*	ATU1, ALT1, ALT2, ALT3 [25,26]	BTU1, BTU2, BLT1, BLT2, BLT3, BLT4, BLT5, BLT6 [25,26]

**Table 2:** Chromosome location and function of different enzymes involved in different tubulin PTMs. (Refer to https://omim.org/ (accessed on 29 October 2025) for the chromosome locations).

Tubulin PTM	Enzyme(s) Involved	Sites of PTMs	Main Cellular Functions	Chromosome Location (Human)
**Acetylation**	αTAT1 (MEC17), San acetyltransferase	α-tubulin: K40, β-tubulin: K252	Increases microtubule flexibility and stability, enhances kinesin-1 and dynein motility; protects lattice from mechanical stress [54]	**6p21.33**
**Methylation**	SET8, SETD2	α-tubulin: K40, K311; β-tubulin: K19, K297	Modulates MT stability; affects mitotic spindle assembly [55,56]	SET8- **12q24.31**
**Palmitoylation**	Palmitoyl-S-acyl-transferases	α-tubulin: C376	Affects membrane-associated trafficking [57]	Multiple loci
**Polyamination**	Transglutaminase 2 (TG2)	α-tubulin: Q31, Q128, Q133, Q285 β-tubulin: Q15	Increases MT resistance to mechanical stress; stabilizes axonal MTs; supports neuronal integrity under load [58,59].	TG2-**20q11.23**
**Glutamylation**	TTLL1, TTLL4, TTLL5, TTLL6, TTLL7, TTLL11, TTLL13	C-terminal Glu residues	Regulates MAP/motor binding; controls severing; essential for cilia beating; modulates axonal [60,61,62] & mitochondrial transport [63,64]	TTLL4-**2q35**, TTLL5-**14q24.3**
**Glycylation**	TTLL3, TTLL8, TTLL10	C-terminal Glu residues	Maintains cilia stability; regulates axonemal dynein function; required for axoneme assembly; affects MT surface diffusion [65,66,67]	TTLL3-**3p25.3**, TTLL8-**22q13.33**
**Tyrosination**	TTL	C-terminal Tyr of α-tubulin	Marks dynamic microtubules and promotes dynein initiation, retrograde IFT, and accurate kinetochore–MT attachments [68,69,70]	TTL-**2q14.1**
**Phosphorylation**	Cdk1, Jak2, PKC, CK2, CaMKII, Syk, Fes, Src	α-tubulin: S165,Y432; β-tubulin: S172; γ-tubulin: S385	Regulates spindle assembly and mitotic dynamics; modulates MAP binding and MT polymerization; tunes MT behaviour in response to signalling [49,71,72]	Cdk1-**10q21.2**
**SUMOylation**	SUMO E1/E2/E3 ligases	α-tubulin: K96, K166, K304	Controls tubulin stability and turnover, contributes to cytoskeletal reorganization during mitosis, and fine-tunes tubulin–MAP interactions [73,74,75], and septins.	SUMO1-**2q33.1**
**Ubiquitination**	E3 ligases	α-tubulin: K308; γ-tubulin: K48, K344	Tags tubulin for degradation, involved in chromosome segregation during cell division [76,77,78]	Multiple loci

## Data Availability

No new data were created or analyzed in this study.

## Abbreviations

The following abbreviations are used in this manuscript:

MT	Microtubule
GTP	Guanosine Triphosphate
CTT	Carboxy Terminal Tail
PTM	Post Translational Modification
MAP	Microtubule-Associated Protein
TTL	Tubulin Tyrosine Ligase
TTLL	Tubulin Tyrosine Ligase-Like
CCP	Cytosolic Carboxy Peptidase
IFT	Intraflagellar transport
